# Oxidative Stress and Risk Factors in Adult Patients with Bronchial Asthma: A Clinical Analysis of Representative Biomarkers

**DOI:** 10.3390/jcm14114007

**Published:** 2025-06-05

**Authors:** Oana-Elena Melinte, Emanuel Ioan Stavarache, Mona Elisabeta Dobrin, Andrei Tudor Cernomaz, Ionel-Bogdan Cioroiu, Daniela Robu Popa, Ionela-Alina Grosu-Creanga, Andreea Zabara Antal, Antigona Carmen Trofor

**Affiliations:** 1 Discipline of Pneumology, III-rd Medical Department, Faculty of Medicine, “Grigore T. Popa” University of Medicine and Pharmacy, 700115 Iasi, Romania; oana-elena.melinte@umfiasi.ro (O.-E.M.); tudor.cernomaz@umfiasi.ro (A.T.C.); daniela.robu-popa@d.umfiasi.ro (D.R.P.); ionela.grossu@yahoo.com (I.-A.G.-C.); andreeazabara@yahoo.com (A.Z.A.); antigona.trofor@umfiasi.ro (A.C.T.); 2 Biochemistry Department, Clinical Hospital of Pulmonary Diseases, 700116 Iasi, Romania; elisabeta-mona.dobrin@pneumo-iasi.ro; 3 Romanian Academy-Iasi Branch, Research Center for Oenology, 700490 Iasi, Romania; bogdan.cioroiu@acadiasi.ro

**Keywords:** oxidative stress, asthma, malondialdehyde, glutathione

## Abstract

**Background:** Asthma is a chronic inflammatory airway disease in which oxidative stress and antioxidant imbalance play a critical role in disease progression and therapeutic response. This study aimed to evaluate oxidative stress and antioxidant status in relation to asthma control levels. **Methods**: A total of 106 patients admitted to the Clinical Hospital of Pulmonary Diseases, Iași, between March and May 2024 were included in this study. Patients were classified into three groups based on asthma control: well-controlled (AB-TCG), partially controlled (AB-PCG), and uncontrolled asthma (AB-UCG). Demographic, biochemical, and hematological parameters were assessed, with attention to oxidative stress markers and antioxidant defenses. **Results**: The study population was predominantly female (75%), with a mean age ranging from 50.75 to 64.38 years, and the majority residing in rural areas (73–75%). The AB-UCG group showed significantly elevated inflammatory markers, including a white blood cell count of 9.33 × 10^3^/µL (*p* = 0.005) and eosinophil percentage of 4.20% (*p* = 0.03), compared with the other groups. This group also exhibited an unfavorable lipid profile, with increased total cholesterol (207.40 mg/dL) and triglyceride levels (157.21 mg/dL). Oxidative stress was notably higher in the AB-UCG group, as indicated by elevated malondialdehyde (MDA) levels (2.86 mmol/L) versus 2.35 mmol/L in the AB-PCG group (*p* < 0.005), along with decreased serum uric acid (4.64 mg/dL) and reduced glutathione (GSH) levels (275.41 µmol/L), leading to a lower GSH/GSSG ratio. Environmental exposures, including tobacco smoke and occupational chemicals, were associated with exacerbated oxidative imbalance. **Conclusions:** The findings highlight the critical involvement of oxidative stress and compromised antioxidant defenses in poorly controlled asthma. Biomarkers such as MDA, white blood cell count, eosinophil percentage, and the GSH/GSSG ratio may act as valuable tools for personalized asthma management and therapeutic monitoring.

## 1. Introduction

Bronchial asthma is a common chronic respiratory condition marked by significant clinical variability, characterized by inflammation and reversible airway obstruction, leading to symptoms such as breathing difficulties, coughing, wheezing, and chest tightness [1,2]. Affecting both children and adults, its rising global prevalence has made it a major public health concern, with approximately 265 million individuals affected worldwide [3,4]. This increase is particularly evident in urban areas, where air pollution and urban allergens contribute significantly to asthma’s progression. The origins of asthma are multifactorial, involving genetic predispositions and environmental factors, such as exposure to allergens (e.g., dust mites, pollen, and mold), air pollution, passive smoking, and viral respiratory infections [5,6]. A critical factor in asthma pathogenesis is oxidative stress, which plays a major role in airway inflammation [6,7,8]. This inflammation leads to vascular hyperreactivity, airway edema, remodeling, and bronchoconstriction, contributing to episodes of airway obstruction [9,10]. Oxidative stress arises from the sustained activation of immune cells within the airways of asthmatic individuals, leading to increased pulmonary levels of reactive oxygen species (ROS). These elevated ROS levels can impact mucus secretion and alter the capillary endothelium, allowing ROS to enter systemic circulation, thereby exacerbating inflammation and tissue damage [11,12,13,14].

While regular pharmacological treatments, such as inhaled corticosteroids and bronchodilators, are effective in managing inflammation and preventing exacerbations in well-controlled asthma [15], residual inflammation and oxidative stress may persist, particularly during exposure to asthma triggers [16]. Oxidative stress plays a key role in asthma exacerbations, contributing to airway inflammation and damage to respiratory tissues, potentially worsening symptoms in uncontrolled asthma [17].

Several mechanisms contribute to oxidative stress in asthma, including excessive ROS production, impaired airway epithelial cell function, chronic inflammation, and airway remodeling. ROS are generated through cellular metabolism and by inflammatory cells such as neutrophils, eosinophils, macrophages, and T lymphocytes that infiltrate the airways [18]. Furthermore, exposure to pollutants and allergens further stimulates ROS production. Numerous studies have investigated the relationship between inflammation and oxidative stress in asthma progression [19,20,21], and examined oxidative stress biomarkers in relation to asthma control. They found that patients with uncontrolled asthma had higher levels of oxidative stress markers and lower levels of some antioxidants compared with those with controlled asthma [22].

In particular, biomarkers of oxidative stress, such as malondialdehyde (MDA)—a marker of lipid peroxidation—are commonly studied [23]. Additionally, components of the antioxidant system, including reduced glutathione (GSH), oxidized glutathione (GSSG), and the GSH/GSSG ratio, are used to assess oxidative stress status in asthma [24,25]. MDA is a stable product of fatty acid peroxidation and is frequently used as a biomarker in clinical studies [26]. The GSH/GSSG ratio, with a normal ratio of approximately 10:1 in healthy individuals, is another important marker; deviations from this ratio indicate oxidative stress [27].

Recent studies have explored the relationship between oxidative stress markers (MDA) and antioxidant enzymes depending on the level of control of bronchial asthma. The plasma levels of MDA are significantly elevated in uncontrolled asthma, suggesting that it may act as a predictive biomarker for asthma control, in line with previous research that identified the serum MDA concentration as a key marker of asthma severity [23].

The aim of the present study is to evaluate antioxidant status in asthma patients and its correlation with asthma control levels. A key objective is to identify potential risk factors by correlating clinical markers of oxidative stress—reduced glutathione, the GSH/GSSG ratio, and MDA—with patient histories of smoking and occupational exposures.

## 2. Materials and Methods

The study population included 53 patients diagnosed with bronchial asthma and 53 patients without bronchial asthma, who constituted the control group.

Among the patients with bronchial asthma, subgroups were identified based on therapeutic control: patients with well-controlled asthma (AB-TCG), partially controlled asthma (AB-PCG), and uncontrolled asthma (AB-UCG). All patients were evaluated throughout their hospitalization. To classify the patients, we used the Asthma Control Test (ACT), a validated and frequently used instrument to assess asthma control based on patient symptoms and medication use. ACT scores correlate with clinical asthma control and allow for the classification of patients into different control categories. Thus, patients with ACT scores = 25 were included in the totally controlled therapeutic asthma (AB-TCG) subgroup, those with scores between 20 and 24 were classified into the partially controlled therapeutic asthma (AB-PCG) subgroup, and patients with ACT scores < 20 were classified into the poorly controlled (AB-UCG) subgroup. In addition to ACT scores, we also considered relevant clinical and laboratory parameters for assessing the patients’ status during hospitalization. These included: evaluation of the frequency and intensity of symptoms, such as cough, wheezing, dyspnea, and the use of rescue medication (short-acting bronchodilators); measurements of pulmonary function, including spirometry, to assess the reversibility of airway obstruction; and determination of inflammatory markers, such as eosinophilia, which can reflect airway inflammation. These combined assessments allowed us to obtain a comprehensive picture of asthma control and to classify patients appropriately.

### 2.1. Patient Eligibility and Management

This study included asthma patients with various comorbidities, such as hypertension, diabetes, obesity, depressive disorder, hypothyroidism, cardiac conditions, hypercholesterolemia, and osteoporosis. Patients in the control group generally had associated comorbidities, such as hypertension, diabetes, obesity, and cardiac conditions (Table 1).

#### 2.1.1. Inclusion Criteria

The inclusion criteria for this study focus on specific patient characteristics essential for evaluating asthma treatment efficacy. Patients must have been diagnosed with asthma for at least six months, ensuring chronicity and stability in their condition. The diagnosis of asthma was confirmed based on clinical symptoms, patient history, and spirometry results, which showed reversible airway obstruction after the administration of short-acting bronchodilators. Additionally, to exclude other conditions that could present with symptoms similar to asthma (e.g., chronic obstructive pulmonary disease—COPD), all patients were evaluated for respiratory comorbidities, and the diagnosis of asthma was confirmed based on the diagnostic criteria of the Global Initiative for Asthma (GINA) 2024 guidelines. They must also have been over 18 years old, which aligns with adult asthma management guidelines. Furthermore, the requirement for treatment with inhaled corticosteroids (ICS) and either oral or inhaled beta2-agonists indicates that participants are receiving standard asthma therapy, allowing for a more accurate assessment of treatment outcomes.

Regarding the control group, we used a strict set of criteria to ensure that participants did not suffer from asthma. They were evaluated through detailed medical history and underwent normal respiratory functional tests, which did not identify obstructive ventilatory dysfunction. In addition, they had no clinical symptoms of asthma, such as coughing, wheezing, or shortness of breath. We considered these procedures sufficient to exclude asthma from the control group and to ensure the validity of the comparisons between the two groups.

#### 2.1.2. Exclusion Criteria

Exclusion criteria consisted of patients diagnosed with respiratory infections or immune deficiencies requiring specific therapy, or any other diseases that may affect the progression of asthma. Patients diagnosed with COPD or cystic fibrosis, pregnant women, those with severe cardiovascular diseases, and individuals with cancer or recent cancer treatment were excluded from this study.

#### 2.1.3. Baseline Characteristics of the Study Population

The demographic data of the investigated patients included age, sex, residence (urban/rural), smoking history, and occupational exposure (Table 1). Epidemiological characteristics, clinical symptoms, and laboratory paraclinical investigations were obtained from the hospital’s electronic system. Laboratory paraclinical evaluations included biochemical parameters such as alanine aminotransferase (ALT/TGP), aspartate aminotransferase (AST/TGO), urea, creatinine, glucose, uric acid, C-reactive protein (CRP), total cholesterol, triglycerides, and complete blood count. Additionally, oxidative stress markers were assessed for all patients included in this study, such as reduced glutathione (GSH), glutathione disulfide (GSSG), the GSH/GSSG ratio, and malondialdehyde (MDA). This study aimed to evaluate oxidative stress markers based on the therapeutic control of asthma patients, as well as to quantify the antioxidant status in relation to tobacco smoke exposure and occupational exposure.

### 2.2. Methods of Analysis

#### 2.2.1. Determination of Biochemical Parameters

Biochemical parameter quantification was performed using the Cobas Integra 400 plus automated analyzer (Roche Diagnostics, Mannheim, Germany). All tests were carried out within the Biochemistry Laboratory following the quality assurance procedures, including both internal quality control and external quality control.

#### 2.2.2. Determination of Hematological Parameters

Hematological parameter quantification in patients with asthma was performed using flow cytometry with the automated analyzer Sysmex (Sysmex Corporation, Kobe, Japan). A CBC (complete blood count) was performed, and the following hematological parameters were evaluated in the present study: white blood cells (WBCs), red blood cells (RBCs), hemoglobin (HGB), hematocrit (HCT), platelets (PLT), lymphocytes (L), monocytes (M), eosinophils (EO), and neutrophils (N).

#### 2.2.3. Quantification of Glutathione in Blood Samples

The described method for determining glutathione in plasma applies high-performance liquid chromatography (HPLC) with precolumn derivatization using o-phthalaldehyde (OPA). Total, oxidized, and protein-bound glutathione is measured after reduction with mercaptoethanol and protein precipitation with perchloric acid. The oxidized forms of glutathione are assessed by blocking free sulfhydryl groups with N-ethylmaleimide (NEM). Reduced glutathione is determined by differentiating between total and oxidized glutathione [28]. The instruments used include a Shimadzu Nexera X2 liquid chromatograph (Shimadzu Corporation, Kyoto, Japan) equipped with a fluorescence detector and a Kinetex C18 XB column (Phenomenex Inc., Torrance, CA, USA). Precolumn derivatization is performed offline, with the sample and derivatization reagent mixed prior to injection. The mobile phase consists of an isocratic mixture of acetate buffer and acetonitrile, with gradient elution. This method allows for the precise measurement of glutathione forms in human blood.

#### 2.2.4. Quantification of Malondialdehyde

The described method for determining malondialdehyde (MDA) is based on high-performance liquid chromatography (HPLC) coupled with mass spectrometry (LC-MS) [26]. The MDA quantification method involves derivatization with 3-nitrophenylhydrazine hydrochloride (3NPHHCl), followed by analysis using HPLC with LC-MS detection. The derived MDA solutions were prepared at different concentrations, and human plasma samples underwent chemical treatment, derivatization, and analysis for MDA quantification. The internal standard used was ^13^C_6_-3NPHHCl.

### 2.3. Statistical Data Analysis

Descriptive statistics for the paraclinical constants were expressed as means, standard deviations, as well as minimum and maximum values. Differences between the analyzed groups (patients with well-controlled asthma, partially controlled asthma, and uncontrolled asthma) were evaluated using the non-parametric Mann–Whitney U test. To identify correlations between the biochemical and hematological markers of the subjects investigated, non-parametric Spearman tests were conducted. To compare these variables across the three asthma control subgroups (AB-TCG, AB-PCG, and AB-UCG), a non-parametric test for comparing multiple groups, such as the Kruskal–Wallis test, was applied. Risk factors associated with disease severity were analyzed using logistic regression with the Statistica 10 software. Additionally, discriminant analysis was used to identify significant predictors for differentiating the studied patient groups.

### 2.4. Ethics Consideration

This study was approved by the ethics committee of the Clinical Hospital of Pulmonary Diseases, Iasi, Romania (ethical approval no.96/16 March 2023) and by the ethics committee of the University of Medicine and Pharmacy “Grigore T. Popa” Iasi, Romania (ethical approval no 319/30 May 2023).

## 3. Results

A total of 53 patients diagnosed with bronchial asthma and multiple comorbidities were investigated at the Iași Clinical Hospital of Pulmonary Diseases between March and May 2024. The demographic data of the patients are presented in Table 1. The patient study population was divided into three groups: patients with well-controlled asthma (n = 12), patients with partially controlled asthma (n = 18), and patients with uncontrolled asthma (n = 23) (Table 2). The demographic data show an average age range of patients between 50.75 and 64.38 years, with a predominance of females (75%) in the three groups investigated. The demographic status of the patients shows a predominance of rural residents (73–75%), especially among patients with partially controlled asthma and those with uncontrolled asthma. Regarding tobacco smoke exposure, there was a predominance of non-smokers (60–75%) in all three groups, while smokers were more frequently found in the uncontrolled asthma group (30%) (Table 1). Patient interviews revealed professional exposure to harmful substances (textile industry, detergent industry, foundry, and chemical industry) among patients in the uncontrolled asthma group (70%), while exposure in the controlled and partially controlled asthma groups was between 50 and 55% (Table 1).

### 3.1. Biological Variables Profile in Asthma Patients

The results of descriptive statistics for biochemical and hematological parameters in patients diagnosed with bronchial asthma are presented in Table 2. It is observed that the mean values for the biological variables of the investigated patients are within the reference biological range, with slight modifications regarding the CRP concentrations for the group of patients with partially controlled asthma (CRP = 9 mg/L). Regarding hematological parameters, the descriptive analysis revealed higher values for white blood cell count (WBC = 9.33 × 10^3^/µL) and eosinophil percentage (4.20%) in the group of patients with uncontrolled asthma (AB-UCG). Patients with uncontrolled asthma exhibited a biochemical profile characterized by elevated serum concentrations of total cholesterol (207.40 mg/dL), triglycerides (157.21 mg/dL), and glucose (6.22 mmol/L) (Table 2).

In this study, the statistical differences between patients with well-controlled asthma (AB-TCG), partially controlled asthma (AB-PCG), and uncontrolled asthma (AB-UCG) were evaluated using the Mann–Whitney U test. Statistically significant differences were observed for white blood cell count (WBC) (*p* = 0.005) and eosinophil count (*p* = 0.03). The comparative study between the three groups also highlighted a statistically significant difference for malondialdehyde (MDA), especially between patients with AB-UCG and AB-PCG (*p* = 0.0009), as well as for those in the AB-TCG group (*p* = 0.005) (Table 3).

### 3.2. Biochemical Profile of Oxidative Stress in Asthma Patients

The antioxidant profile was compared in asthma patients based on the three groups: well-controlled asthma (AB-TCG), partially controlled asthma (AB-PCG), and uncontrolled asthma (AB-UCG). Statistical analysis revealed significant differences between the patient groups. Patients in the AB-UCG group showed the lowest mean values for serum uric acid (4.64 mg/dL) and reduced glutathione (275.41 µmol/L) compared with patients in the AB-TCG and AB-PCG groups. Furthermore, increased concentrations of malondialdehyde (MDA) were observed in patients with uncontrolled asthma (2.86 mmol/L) and partially controlled asthma (2.35 mmol/L) (Table 4).

**Figure 1:**
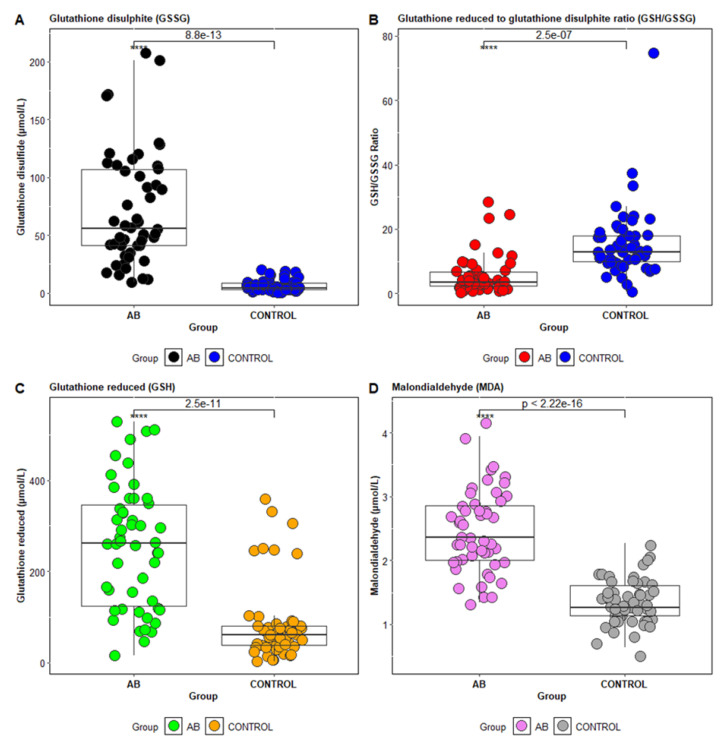
Level of oxidative stress parameters in asthmatic patients (AB) versus healthy controls (CONTROL). (**A**)—Glutathione disulfite, (**B**)—Glutathione reduced to glutathione disulfite ratio (GSH/GSSG). (**C**)—Glutathione reduced (GSH), (**D**)—Malondialdehyde (MDA). Statistical significant differences are indicated as follows: *p* < 0.0001 (****). Observed *p*-values: GSSG (8.8 × 10^−13^), GSH/GSSG (2.5 × 10^−7^), GSH (2.5 × 10^−11^), MDA (<2.22 × 10^−16^).

The distribution of values for MDA, GSH, and GSSG concentrations, and the GSH/GSSG ratio for patients in the control group versus asthma patients is shown in Figure 1A–D). The GSH/GSSG ratio is used as an indicator of cellular redox status, and under physiological conditions, it has a value greater than 9.0 [29,30]. A change in this ratio toward GSSG indicates a high level of free radicals, thus acting as a marker of increased oxidative stress [31].

**Figure 2:**
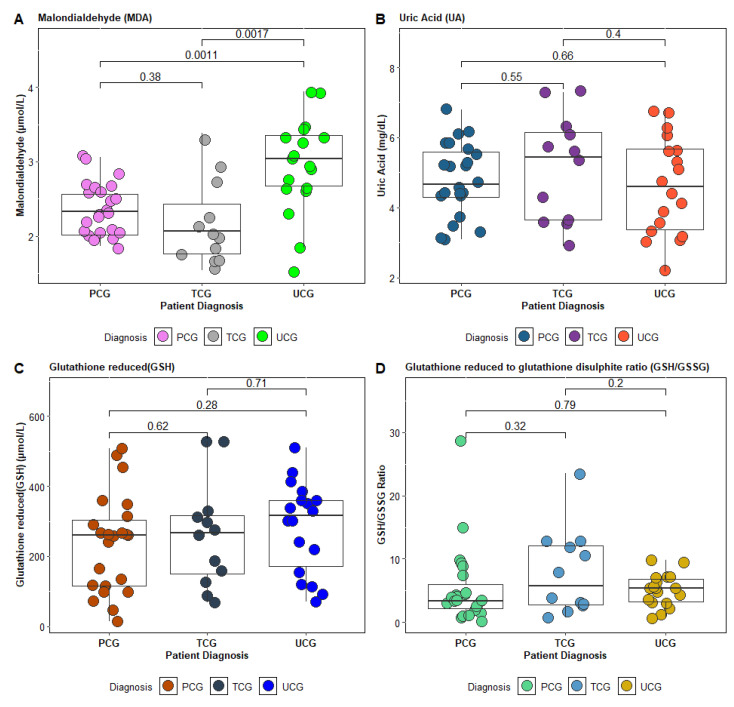
Concentration profile of oxidative stress parameters (**A**)—Malondialdehyde (MDA), (**B**)—Uric acid (UA), (**C**) Glutathione reduced (GSH), (**D**) Glutathione reduced to glutathione disulfite ratio (GSH/GSSG) in total control group (TCG), partial controlled group PCG) versus uncontrolled group (UCG).

The concentrations of oxidative stress markers investigated based on the asthma control level for the patients under study are presented in Figure 2. A statistically significant difference was observed, particularly for MDA (*p* ˂ 0.005), between the AB-UCG and AB-TCG patient groups (Figure 2A).

### 3.3. Biochemical Profile of Oxidative Stress in Asthmatic Patients with Tobacco Smoke Exposure

Increased oxidative stress is a clinical characteristic of asthma that promotes inflammatory responses in the bronchial epithelial cells of the respiratory airways. It has been observed that both smokers and non-smokers with asthma exhibit alterations in markers of oxidative stress [32].

This study evaluated oxidative stress markers in asthmatic patients, categorizing them based on smoking status. Statistical results showed lower serum uric acid levels in smokers (5.29 mg/dL) and former smokers (4.75 mg/dL) compared with non-smokers (5.76 mg/dL). The plasma concentrations of reduced glutathione (GSH) were also lower in former smokers (232.90 µmol/L) and non-smokers (249.77 µmol/L). Interestingly, the GSH/GSSG ratio was higher in smokers (7.79) than in former smokers (3.94) and non-smokers (5.45). The MDA concentrations were the highest in former smokers with asthma (2.70 mmol/L), compared with smokers (2.47 mmol/L) and non-smokers (2.38 mmol/L) (Table 5).

### 3.4. Biochemical Profile of Oxidative Stress in Asthmatic Patients with Occupational Exposure to Chemical Agents

According to demographic data, a considerable percentage (30–55%) of the asthmatic patients investigated reported occupational exposure to chemical agents, suggesting a significant occupational risk compared with the control group (10%) (Table 1). In the present study, the concentrations of non-enzymatic antioxidants such as serum uric acid, oxidized glutathione (GSSG), reduced glutathione (GSH), and the GSH/GSSG ratio were evaluated in asthmatic patients.

The results of this study highlighted lower serum uric acid values in asthmatic patients with occupational exposure (4.52 mg/dL) as well as lower GSH/GSSG ratios (4.74) compared with patients without reported occupational exposure to harmful substances (5.71) (Table 6). The average concentration of plasma MDA was 2.56 mmol/L in asthmatic patients with occupational exposure, with values ranging from 1.41 to 3.94 mmol/L.

**Figure 3:**
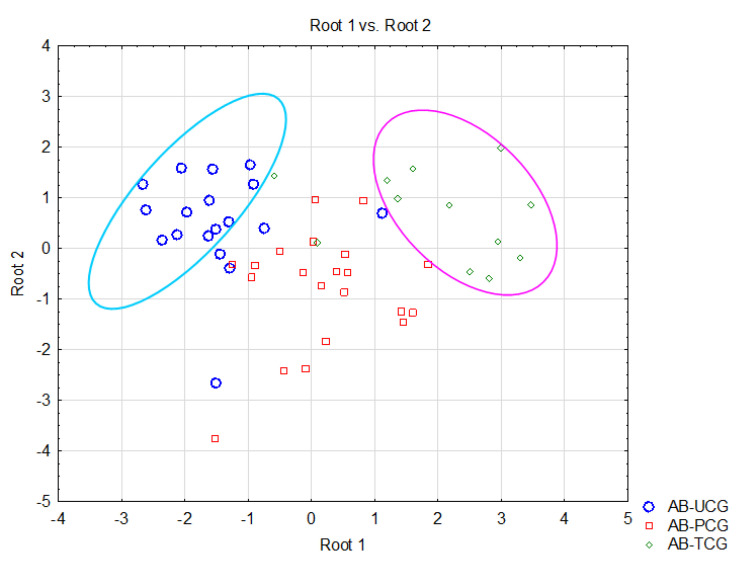
Representation of scores linked to clinical parameters according to the asthma control levels (AB-TCG—Therapeutically controlled asthma group; AB-PCG—Asthma partial therapeutic control group; AB-UCG—Asthma uncontrolled therapeutic group).

Thus, this study aimed to identify biological variables that could differentiate the investigated patients based on their level of asthma control. The discriminant analysis applied to the dataset highlighted five parameters that significantly contributed to the separation of the considered patient classes. Table 7 presents the results regarding the selection of the most important statistical variables. These results show that, out of the 22 parameters analyzed, only 5 were retained as representative for the model, meaning that they significantly contributed to the separation of the proposed classes for this study, namely AB-UCG, AB-PCG, and AB-TCG. Specifically, the concentrations of MDA (*p* = 0.000), the number of WBCs (*p* = 0.000), the number of eosinophils (*p* = 0.002), the number of RBCs (0.007), and the GSH/GSSG ratio values (*p* = 0.002) were the parameters with the highest importance in separating the considered classes (Table 7). Figure 3 illustrates the classes and the relationships between them by showing the individual scores of the investigated subjects along the main discriminant functions.

## 4. Discussion

The aim of the present study was to assess oxidative stress and antioxidant status in relation to asthma control levels in 106 patients enrolled at the Clinical Hospital of Pulmonary Diseases, Iasi. The study population included patients with controlled asthma, partially controlled asthma, and uncontrolled asthma. Biochemical and hematological parameters were measured, and statistical analysis of the three patient groups revealed significant differences (*p* < 0.005), particularly in the white blood cell (WBC) count. The literature indicates that individuals with asthma typically exhibit elevated WBC levels in both peripheral blood and airway secretions compared with non-asthmatic individuals. Increased WBC levels are considered a marker of inflammation and are implicated in the pathophysiology of asthma symptoms [33]. Inflammatory cells, such as eosinophils recruited into the asthmatic airway, possess a remarkable capacity for reactive oxygen species (ROS) production. Once recruited, these cells are activated and generate ROS in response to various stimuli [34]. In this study, significant differences in eosinophil counts were particularly evident in patients with uncontrolled asthma. The respiratory medicine literature suggests that the activation of eosinophils, neutrophils, and macrophages can result in substantial oxidative stress through ROS production [35,36].

The lipid profile in patients with uncontrolled asthma may exhibit distinct characteristics, primarily due to chronic inflammation and metabolic disturbances associated with severe asthma. Uncontrolled asthma is frequently marked by recurrent exacerbations and necessitates more aggressive treatment regimens. Assessing the lipid profile in asthma patients is crucial not only for identifying cardiovascular risks, but also for optimizing treatment strategies. Monitoring total cholesterol and triglyceride levels is especially important in the context of long-term corticosteroid therapy, which can significantly affect lipid profiles. Recent studies have found a positive association between serum triglycerides, total cholesterol, and asthma, highlighting the potential implications of lipid levels in asthma pathophysiology and clinical management [37]. Various factors—including chronic inflammation, medications, and lifestyle—can modulate the lipid profile in asthma. Regular monitoring of lipid parameters is essential to better understand cardiovascular risks and any adverse effects of asthma treatments [38].

Such biomarkers could serve as valuable tools for assessing and monitoring asthma severity and treatment response. Prior studies have shown that elevated leukocyte counts correlate with asthma severity, and increased MDA levels are indicative of enhanced oxidative stress, both contributing to asthma progression [39,40]. Discriminant analysis applied to the asthma patients in this study revealed notable homogeneity within the dataset, particularly among the AB-UCG and AB-TCG groups (Figure 3). The concentration of MDA, WBC count, eosinophil count, RBC count, and the GSH/GSSG ratio emerged as the most significant predictors for distinguishing between the patient groups. These results suggest that oxidative stress and inflammation—measured via MDA, WBC, E%, and GSH/GSSG—could serve as critical biomarkers for clinically differentiating asthma patients based on therapeutic control.

It is well-established that persistent chronic inflammation is a key element in the pathogenesis of bronchial asthma, triggered by a combination of biological, chemical, and physical factors. These factors, together with individual and environmental risk factors, contribute to an increased likelihood of disease onset. Inflammatory responses lead to the generation of reactive oxygen species (ROS), and prolonged exposure to ROS induces oxidative stress, which in turn amplifies immune responses and the production of inflammatory mediators [41].

In this study, antioxidant status was evaluated by measuring the serum uric acid and glutathione levels in the blood of the investigated patients. The findings revealed significantly lower serum uric acid concentrations and a reduced GSH/GSSG ratio, especially in patients with uncontrolled asthma.

Therapeutic control of asthma significantly influenced the antioxidant profile of the patients: those with uncontrolled asthma exhibited an altered antioxidant status, characterized by low serum uric acid and reduced glutathione (GSH) levels, along with elevated malondialdehyde (MDA) levels.

The decrease in GSH levels in asthma patients may be caused either by increased consumption of GSH or by a deficiency in the amino acids required for glutathione synthesis. A study observed decreased levels of glycine and glutamic acid in children with asthma, suggesting that this decrease might result from the excessive use of these amino acids to support the increased production of glutathione in an attempt to combat free radicals [22].

As asthma control improved, these marker values approached those of the control group [42]. Although the asthmatic patients in this study did not exhibit overt respiratory symptoms, the findings suggest that even well-controlled asthma patients may experience substantial oxidative stress (reflected by a GSH/GSSG ratio <9), which could be a risk factor for future exacerbations or complications. Other studies focusing on poorly controlled asthmatics with marked airway inflammation have similarly reported lower systemic GSH concentrations [43]. Although the exact mechanisms underlying variations in GSH concentrations in asthma remain under investigation, numerous studies have explored reductions in GSH levels and related factors in both the respiratory tract and systemic circulation of asthma patients [44].

This study also evaluated the antioxidant status of asthma patients in relation to tobacco smoke exposure (Table 5) and occupational exposure to reactive oxygen species (ROS) (Table 6). The results revealed lower GSH/GSSG ratios in former and current smokers, as well as in individuals with occupational ROS exposure. Previous research has shown that lung damage is mediated by oxidants and free radicals present in cigarette smoke, released by activated neutrophils. Studies have also demonstrated that cigarette smoke contains a range of toxic chemical compounds that activate neutrophils, leading to the release of ROS and free radicals. These reactive species contribute to oxidative stress, promote tissue damage, and induce chronic inflammation, potentially culminating in severe respiratory diseases such as chronic obstructive pulmonary disease (COPD) and emphysema [45]. The reduction in GSH concentrations caused by cigarette smoke exposure results in increased lipid peroxidation and the activation of genes coding for pro-inflammatory cytokines involved in the pathogenesis of chronic obstructive pulmonary diseases [46]. Also, chronic exposure to toxic compounds in cigarette smoke not only exacerbates respiratory inflammation, but also accelerates the degradation of glutathione, an essential antioxidant in pulmonary defense [47]. This reduction in GSH may contribute to the increased vulnerability of smokers or former smokers to asthma exacerbations and other respiratory comorbidities.

The results suggest that smoking history should be considered an important factor in assessing oxidative stress risk and in adapting therapeutic strategies for asthma patients. In this regard, personalized management approaches could include close monitoring of redox markers, as well as specific recommendations for reducing exposure to pro-oxidative environmental factors. Comparing these results with previous studies supports the validity of the conclusions and opens new directions for further research to investigate the molecular mechanisms of redox imbalances in asthma.

## 5. Conclusions

This study highlights the significant role of oxidative stress in bronchial asthma, particularly in patients with uncontrolled disease. Systemic inflammation, indicated by leukocytosis and eosinophilia, is closely associated with increased oxidative stress markers, such as MDA, and altered lipid profiles, emphasizing the link between chronic inflammation, metabolic dysfunction, and cardiovascular risk. Exposure to tobacco smoke and occupational chemicals further exacerbates oxidative stress, reflected by low GSH/GSSG ratios. These findings suggest the importance of monitoring oxidative stress markers and implementing strategies to reduce environmental and occupational triggers. This study also shows that biomarkers like MDA, WBC count, eosinophil percentage, and GSH/GSSG ratios are valuable for assessing asthma control and may serve as clinical tools for monitoring disease progression and response to therapy. Future research should continue to investigate the mechanisms driving these alterations and evaluate the potential benefits of antioxidant therapies in mitigating asthma-related oxidative stress.

### Limitations

Future studies should prioritize larger patient samples to identify the most relevant oxidative stress markers for asthma control. Additionally, research should focus on local oxidative stress in the airways and explore the roles of antioxidants and specific asthma phenotypes to better understand the relationship between oxidative stress and bronchial asthma.

## Figures and Tables

**Table 1:** Demographic and clinical characteristics of the investigated subjects (AB-TCG—Therapeutically controlled asthma group; AB-PCG—Asthma partial therapeutic control group; AB-UCG—Asthma uncontrolled therapeutic group).

	Controls	AB-TCG	AB-PCG	AB-UCG	*p*-Value
	**(n = 53)**	**(n = 12)**	**(n = 18)**	**(n = 23)**	
**Variabiles**					
Age (Mean ± Stdev)	56 ± 15.56	50.75 ± 14.92	64.38 ± 13.31	55.65 ± 15.5	0.50
**Gendre**					
Male (%)	40%	25%	17%	25%	0.64
Female (%)	60%	75%	83%	75%	0.60
**Residential area**					
Urban (%)	59%	50%	27%	25%	0.24
Rural (%)	41%	50%	73%	75%	0.25
**History of smoking**					
Smokers	23%	10%	8%	30%	0.28
Non-smokers	62%	75%	77%	60%	0.47
Ex-smokers	15%	15%	15%	10%	0.30
**Profesional exposure** (Yes/No)					
	10%/90%	50%/50%	55%/45%	70%/30%	0.58
**Internal medicine comorbidities**	**Controls**		**Asthma group**	***p*-value**	
Diabetes mellitus	4 (8%)		7 (13%)	0.002	
Hypertension	17 (32%)		21 (40%)	0.002	
Hypercholesterolemia	3 (6%)		7 (13%)	0.246	
Obesity	6 (11%)		5 (9%)	0.520	
Chronic coronary syndrome	6 (11%)		2 (4%)	0.431	
Osteoporosis	0		6 (11%)	0.581	
Hypothyroidism	0		5 (9%)	0.871	
Depressive disorder	0		6 (11%)	0.562	

**Table 2:** Concentration profile of paraclinical investigation in asthma patients and control group (AB-TCG—Therapeutically controlled asthma group; AB-PCG—Asthma partial therapeutic control group; AB-UCG—Asthma uncontrolled therapeutic group).

	Controls			AB-PCG			AB-UCG			AB-TCG		
Biochemical Parameters	Mean	Stdev	Range	Mean	Stdev	Range	Mean	Stdev	Range	Mean	Stdev	Range
TGP	21.22	12.72	7.9–66.70	18.53	8.92	9.8–46.30	23.04	16.41	7.9–66.70	25.46	14.51	11.7–46.20
TGO	23.32	12.00	11.6–72.10	23.72	12.86	14.6–72.10	22.83	12.47	11.6–52.60	25.99	12.53	14.1–50.10
Uree	31.98	9.21	14.4–54.60	32.16	8.88	16.7–48.90	30.92	10.12	14.4–54.60	32.53	8.68	17–45.90
Creat	0.73	0.18	0.42–1.21	0.70	0.18	0.42–1.21	0.68	0.14	0.46–0.98	0.80	0.19	0.51–1.12
Gluc	5.95	1.22	4.05–10.04	5.83	1.10	4.05–8.28	6.22	1.60	4.24–10.04	5.59	0.84	4.60–7.00
CRP	7.00	12.60	0.6–84.30	9.68	19.17	0.6–84.30	5.33	4.45	0.6–14.30	4.95	6.66	0.9–21.20
Col-T	197.46	47.04	97.64–297.80	196.10	40.47	109.94–258.50	207.40	45.03	140.7–297.80	189.70	67.76	97.64–289
Trigl	143.42	67.47	43.43–315.80	135.97	65.08	59.2–306.86	157.21	81.30	45–315.80	142.30	61.61	43.43–254
**Hematological Parameters**	**Mean**	**Stdev**	**Range**	**Mean**	**Stdev**	**Range**	**Mean**	**Stdev**	**Range**	**Mean**	**Stdev**	**Range**
WBC	8.36	2.68	4.04–16.99	8.32	2.45	4.04–14.70	9.33	3.19	5.85–16.99	6.42	1.36	5.01–9.37
RBC	4.57	0.53	3.15–5.70	4.38	0.50	3.15–5.09	4.62	0.55	3.89–5.70	4.81	0.53	3.97–5.59
HGB	13.53	1.57	10.2–17.20	13.24	1.38	10.5–15	13.46	1.89	10.2–16.50	13.88	1.63	11.4–17.20
HCT	40.91	4.12	31.4–49.50	40.15	3.81	32.6–45.5	40.58	4.84	31.4–48.80	42.08	3.85	37.7–49.50
PLT	275.71	68.62	115–515	276.66	77.59	115–515	295.06	65.45	202–478	248.10	54.46	173–350
N%	61.12	8.72	44.7–83	60.69	11.24	44.7–83	61.34	7.12	48.7–74.50	59.67	6.92	45.9–68.70
L%	27.39	6.58	10.4–41.80	27.29	8.18	10.4–39.30	27.08	5.30	13.2–34.70	29.43	6.00	23.8–41.80
M%	8.13	1.80	5.1–13.80	8.25	1.99	5.1–13.60	7.90	1.63	5.6–11.60	8.65	2.07	6.5–13.80
E%	2.84	2.71	0–4.60	3.23	3.51	0–14.6	4.20	2.30	0.2–7.80	1.70	1.71	0–5.40

**Table 3:** Application of the Mann–Whitney U test between the investigated groups.

Mann–Whitney U Test AB-TCG and AB-UCG									
	**Rank Sum—AB-TCG**	Rank Sum—AB-UCG	U	Z	*p*-Value	Z—Adjusted	*p*-Value	Valid N—AB-TCG	Valid N—AB-UCG
**MDA**	123	373	45	−277,804	0.005469	−277,994	0.005446	12	18
**WBC**	124	372	46	−273,749	0.006191	−273,859	0.005171	12	18
**E%**	138	358	60	−216,971	0.030029	−217,564	0.029583	12	18
Mann–Whitney U Test AB-TCG and AB-PCG									
	**Rank Sum—AB-TCG**	**Rank Sum—AB-PCG**	**U**	**Z**	***p*** **-Value**	**Z—Adjusted**	***p*** **-Value**	**Valid N—AB-TCG**	**Valid N—AB-PCG**
**WBC**	148	447	70	−221,631	0.026671	−221,698	0.026625	12	23
**RBC**	262	333	80	185,593	0.063464	185,692	0.063323	12	23
Mann–Whitney U Test AB-UCG and AB-PCG									
	**Rank Sum—AB-UCG**	**Rank Sum—AB-PCG**	**U**	**Z**	***p*** **-Value**	**Z—Adjusted**	***p*** **-Value**	**Valid N—AB-UCG**	**Valid N—AB-PCG**
**MDA**	526	335	82	330,726	0.000942	3,307,692	0.000941	18	23

**Table 4:** Biochemical profile of the redox balance in asthma patients.

	AB-PCG			AB-UCG			AB-TCG			
Parameters	Mean	Stdev	Range	Mean	Stdev	Range	Mean	Stdev	Range	*p*
Uric acid (mg/dL)	4.92	0.99	3.1–6.80	4.64	1.41	2.17–6.80	5.06	1.42	2.9–7.30	0.41
GSSG (µmol/L)	71.27	46.80	9.59–201.13	69.05	48.48	9.596–201.13	73.41	59.83	11.66–207.29	0.005
GSH (µmol/L)	284.18	129.40	69.414–510.6	275.41	130.98	69.41–510.6	299.96	134.36	67.78–528.43	0.004
GSH/GSSG	5.15	2.54	0.59–9.78	5.10	4.22	0.59–24.39	6.54	7.28	0.76–23.52	0.003
MDA (mmol/L)	2.35	0.36	1.87–3.06	2.86	0.77	3/1/1994	2.18	0.54	1.54–3.38	0.005

**Table 5:** Biochemical profile of redox balance based on smoking status in asthmatic patients.

	Non-Smokers (n = 35)			Smokers (n = 9)			Ex-Smokers (n = 6)			
Parameters	Mean	Stdev	Range	Mean	Stdev	Range	Mead	Stdev	Range	*p*
Uric acid (mg/dL)	5.76	1.08	2.9–6.30	5.29	1.86	1.85–5.29	4.75	0.94	3.1–5.67	0.41
GSSG (µmol/L)	78.54	55.06	9.596–207.2	51.97	29.02	29.02–51.97	66.09	20.58	44.85–93.0	0.01
GSH (µmol/L)	249.7	132.4	45.69–510.6	293.86	138.0	138.0–293.8	232.90	161.7	14.76–438.6	0.01
GSH/GSSG	5.45	6.12	0.59–28.59	7.79	7.10	1.715–24.39	3.94	3.36	0.25–9.78	0.01
MDA (mmol/L)	2.38	0.67	1.34–3.94	2.47	0.47	1.79–3.06	2.70	1.01	1.69–4.17	0.00

**Table 6:** Biochemical profile of the redox balance based on professional exposure to patients diagnosed with bronchial asthma.

	Patients With Professional Exposure				Patients Without Professional Exposure			
Parameters	Mean	Stdev	Min	Max	Mean	Stdev	Min	Max
Uric acid (mg/dL)	4.51	0.96	2.90	5.70	5.08	1.33	2.17	7.30
GSSG (µmol/L)	90.07	49.64	11.66	207.29	61.40	43.83	9.60	171.73
GSH (µmol/L)	264.29	131.24	14.77	510.60	248.95	135.85	45.70	528.43
GSH/GSSG	4.74	5.44	0.25	23.52	5.71	4.83	0.67	24.39
MDA (mmol/L)	2.56	0.80	1.41	3.94	2.26	0.81	1.34	4.17

**Table 7:** Summary of the discriminant function analysis for the clinical parameters examined, based on the three cases considered.

Variables	Wilks’	Partial	F-Remove	*p*-Value	Toler.	1-Toler.
MDA	0.406	0.606	13.319	0.000	0.698	0.302
WBC	0.444	0.554	16.482	0.000	0.442	0.558
E%	0.334	0.736	7.363	0.002	0.611	0.389
RBC	0.314	0.784	5.641	0.007	0.487	0.513
GSH/GSSG	0.331	0.743	7.101	0.002	0.430	0.570
CRP	0.262	0.938	1.347	0.271	0.869	0.131
AU	0.258	0.952	1.030	0.366	0.452	0.548
TGP	0.284	0.867	3.140	0.054	0.524	0.476
TRIGL	0.278	0.886	2.643	0.083	0.546	0.454
GSH	0.263	0.936	1.398	0.259	0.456	0.544

## Data Availability

The datasets generated and/or analyzed during the current study are available from the corresponding author on reasonable request.
